# A new strategy for isolating genes controlling dosage compensation in *Drosophila *using a simple epigenetic mosaic eye phenotype

**DOI:** 10.1186/1741-7007-8-80

**Published:** 2010-06-10

**Authors:** Mahalakshmi Prabhakaran, Richard L Kelley

**Affiliations:** 1 Program in Developmental Biology, Baylor College of Medicine, Houston, TX, USA; 2 Department of Molecular and Cellular Biology, Baylor College of Medicine, Houston, TX, USA; 3 Department of Molecular and Human Genetics, Baylor College of Medicine, Houston, TX, USA

## Abstract

**Background:**

The *Drosophila *Male Specific Lethal (MSL) complex contains chromatin modifying enzymes and non-coding *roX *RNA. It paints the male X at hundreds of bands where it acetylates histone H4 at lysine 16. This epigenetic mark increases expression from the single male X chromosome approximately twofold above what gene-specific factors produce from each female X chromosome. This equalises X-linked gene expression between the sexes. Previous screens for components of dosage compensation relied on a distinctive male-specific lethal phenotype.

**Results:**

Here, we report a new strategy relying upon an unusual male-specific mosaic eye pigmentation phenotype produced when the MSL complex acts upon autosomal *roX1 *transgenes. Screening the second chromosome identified at least five loci, two of which are previously described components of the MSL complex. We focused our analysis on the modifier alleles of MSL1 and MLE (for 'maleless'). The MSL1 lesions are not simple nulls, but rather alter the PEHE domain that recruits the MSL3 chromodomain and MOF ('males absent on first') histone acetyltransferase subunits to the complex. These mutants are compromised in their ability to recruit MSL3 and MOF, dosage compensate the X, and support long distance spreading from *roX1 *transgenes. Yet, paradoxically, they were isolated because they somehow increase MSL complex activity immediately around *roX1 *transgenes in combination with wild-type MSL1 subunits.

**Conclusions:**

We propose that these diverse phenotypes arise from perturbations in assembly of MSL subunits onto nascent *roX *transcripts. This strategy is a promising alternative route for identifying previously unknown components of the dosage compensation pathway and novel alleles of known MSL proteins.

## Background

Some organisms use a genetic mechanism to specify sex [1]. This often leads to degeneration of one homologue of the critical sex-determining chromosome. Consequently, these species evolve a mechanism to survive the otherwise lethal consequences of monosomy for a large chromosome in one sex. In *Drosophila *the primary mechanism of dosage compensation is male-specific hypertranscription of most genes along his single X chromosome to match the RNA output from the two X chromosomes found in females [2-4]. This hypertranscription is mediated by acetylation of histone H4 at K16 [5-7] throughout the entire body of transcribed genes hinting that the underlying mechanism may be an increase in the elongation rate of RNA polymerase across X-linked genes [8-11]. The mechanism by which dosage compensation is limited to males is well understood [12-15], but how X-linked genes are distinguished from autosomal genes is only beginning to come into focus [16-19].

Dosage compensation is carried out by a chromatin remodelling complex composed of large non-coding *roX *(for 'RNA on the X chromosome') RNAs and at least five MSL (for 'male-specific lethal') proteins: MSL1 (scaffold protein), MSL2 (RING finger protein), MSL3 (chromodomain protein), MOF ('males absent on first', histone H4 acetyltransferase) and MLE (for 'maleless', RNA helicase) [20]. A chromatin remodelling complex consisting of MSL1, MSL2, MSL3 and MOF is also found in vertebrates [21,22]. So far no RNA helicase or non-coding RNA has been linked to the vertebrate MSL complex. MSL1 seems particularly important structurally because it contains docking sites for three of the other subunits. A predicted coiled coil near N-terminus binds MSL2 [23,24], and a domain near the C-terminus, containing a PEHE motif, binds MSL3 and MOF [23,25]. The interactions between the C-terminal region of MSL1 with MSL3 and MOF are the focus of this study. Outside these motifs, most of the sequence is poorly conserved even within the genus *Drosophila*. It is not clear how *roX *RNAs interact with the protein subunits although non-sequence-specific RNA interactions have been reported for MOF, MSL3 and MLE [26-28]. The C-terminal region of MSL2 is required for efficient *roX *RNA incorporation into MSL complexes, but it is not clear if this represents direct protein-RNA contacts [29]. The MSL complex is able to spread long distances *in cis *from sites of *roX *RNA synthesis, and this has been taken as evidence that the MSL protein subunits begin assembling onto nascent *roX *transcripts cotranscriptionally [30-33].

The genes encoding the protein components of the complex were discovered because of their distinctive male-specific lethal loss of function phenotype. The two *roX *genes were found in screens for sex-specific transcripts and later found to act in dosage compensation [34,35]. Several groups have studied the biochemical composition of the dosage compensation complex searching for additional components. These reports indicate that there are unlikely to be additional *roX*-like RNA genes [31], but several proteins copurify with the MSL complex including components of the nuclear pore [36,37]. Additionally, general chromatin remodelling proteins such as ISWI, NURF301, JIL-1, HP1, and Su(var)3-7 display complex genetic interactions with the MSL complex [38-43]. However, in each case it is unclear exactly how the different factors normally interact to produce the proper chromatin architecture along the male X.

If any component of the dosage compensation pathway performed additional functions, it may have been overlooked in screens for male-specific lethal mutants. Enhancer-suppressor screens have been highly successful for identifying components of many pathways in flies and other model organisms, but the sex-specific lethality of dosage compensation is not easily incorporated into such a strategy. For instance, an inversion that places the normally euchromatic *white *locus next to centric heterochromatin results in mosaic *white *expression (mosaic eye pigmentation) in both sexes. Many dozens of dominant modifier *Su(var) *mutants have been isolated that alter the eye colour of *In(1) w*^M4 ^flies. These turned out to encode key chromatin modifying enzymes. Here we report a new genetic strategy that exploits epigenetic male-specific mosaic eye colour as a simple phenotypic readout for local MSL activity amenable to modifier screens. We used unusual transgenic stocks in which a *roX1 *transgene had been inserted in random autosomal sites [44]. Consistent with earlier findings, the autosomal *roX1 *transgenes could support dosage compensation of the X even if they were the only source of *roX *RNA [32,34]. The surprising finding was that while most *roX1 *autosomal insertions expressed the adjacent *miniwhite *marker normally to give solid pigmented eye colour in both males and females, roughly 10% to 15% of the random insertions displayed mosaic patches of eye pigment only in males. Many lines show further pairing-dependent silencing of *miniwhite *where homozygous males have less pigmentation than hemizygotes. These unusual stocks had the transgene integrated in a wide variety of sites, and the females in these stocks had either pale eye colour or completely white eyes indicating that the *miniwhite *reporter was epigenetically silenced. The *miniwhite *marker could be activated in females that were supplied with a complete set of dosage compensation components showing that eye colour was a marker for MSL complex activity [44]. In these stocks, the *roX1 *gene nucleated ectopic dosage compensation at the transgene, opening the surrounding chromatin and permitting *miniwhite *expression in patches in the male eye. When these *roX1 *transgenes that have mosaic eye pigmentation were crossed into males deleted for the endogenous *roX1 *and *roX2 *genes, MSL complex spread > 1 Mbp from the autosomal sites of *roX1 *transcription in essentially all cells in larval salivary glands. This was accompanied by a change to solid red eye colour only in males [32]. This shows that subtle changes in the local distribution or activity of the MSL complex can be easily detected by alterations in the epigenetic male mosaic eye pattern long before they affect male viability.

We exploited these unusual transgenics stocks to carry out a dominant modifier screen searching for mutations that alter MSL complex activity enough to detect a change in male eye colour. In this strategy, all flies retain one wild-type allele of the candidate modifier gene, so that if the gene of interest performs essential functions besides dosage compensation, viable animals are still recovered. It is possible to search for mutants that either epigenetically changed from mosaic to solid red (more MSL activity at the *roX1 *transgene) or nearly solid white (less MSL activity). Here, we report the results of the former screen. Of the 13 mutations isolated, 4 mapped to known components of the dosage compensation complex demonstrating the specificity of the screen design. We focused our analysis on the new mutations in *msl1 *because they likely alter complex assembly and spreading by disrupting the interface that binds MSL3 and MOF, two other well characterised subunits of the dosage compensation complex. The new *msl1 *alleles display a complex pattern of gain of function and loss of function when subjected to a battery of assays.

## Results

### A genetic screen for dominant modifiers of dosage compensation

**Figure 1:**
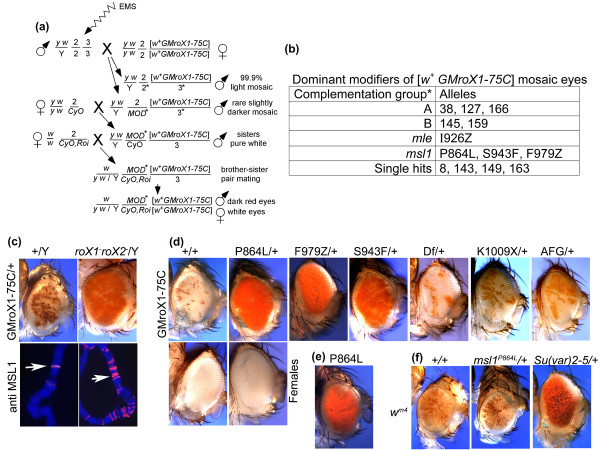
**Modifiers of dosage compensation**. **(a) **From approximately 10,000 G1 sons screened, 13 modifier lines were established. **(b) **The mutations were placed in complementation groups by assaying for recessive lethality. Alleles of groups A and B are indicated by arbitrary isolation numbers and are lethal to both males and females. Alleles of *msl1 *and *mle *are named by their codon changes where Z = frameshift and X = nonsense codon. *The modifiers scored as single hits each carries a recessive lethal mutation, but it is not known whether this maps to the modifier locus or an extraneous gene on the same chromosome. **(c) **The hemizygous *GMroX1*-75C males displays mosaic pigmentation when the endogenous *roX*^+ ^genes are present and the Male Specific Lethal (MSL) complex binds a single band at 75C. Male eye colour is derepressed and MSL complex spreads > 1 Mb when both endogenous *roX *genes are deleted. **(d) **All flies are homozygous for *GMroX1*-75C and heterozygous for the *msl1 *mutation indicated. The top row shows males, the lower two rows are females. The mosaic eyes of *GMroX1-75C *homozygous males become almost solid red when a modifier mutation is present. Mutations *P864L*, *F979Z *and *S943F *are new alleles of *msl1 *that were isolated in the present study. Df = *msl1*^L60 ^[46] has no effect on the mosaic pattern. *AFG *and *K1009X *are previously identified *msl1 *lesions that map near the modifier mutations, but have little or no effect on the mosaic *miniwhite *expression. Bottom rows: *GMroX1-75C *females have pure white eyes and this is not affected by any of the modifier mutations. **(e) **Homozygous P864L escaper males display enhanced *miniwhite *expression. **(f) **The modifier mutants (*msl1^P864L ^*shown) have no effect upon *In(1) w*^m4 ^whereas *Su(var)2-5 *is a potent suppressor of position effect variegation (PEV).

We carried out a dominant F1 enhancer screen (Figure 1a) on a *GMroX1 *transgene that carries a wild-type genomic *melanogaster roX1 *gene on a 4.9 kb *Eco*RI fragment adjacent to the *miniwhite *eye pigmentation marker [45] inserted in a YOYO element at 75C in the euchromatic region of the third chromosome. This transgene is subject to further pairing-dependent silencing in males (compare Figure 1c, d). Initially we found that reducing the dose of the known MSL proteins by half in males heterozygous for *msl1*, *msl2*, *msl3 *and *mle *had no consistent effect on the mosaic eye pattern of males carrying one copy of *GMroX1-75C *(data not shown). However, removing the endogenous *roX1 *and *roX2 *genes resulted in sons with solid red eyes and extensive *cis *spreading of the MSL complex from the transgene, presumably because, in the absence of endogenous roX expression, more MSL subunits are available to associate with the *roX1 *transcripts made at the transgene (Park *et al. *[32] and Figure 1c).

We screened approximately 10^4 ^ethyl methane sulfonate (EMS)-treated sons for mutations that increased the fraction of red eye tissue and established 13 second chromosome mutant stocks that transmitted the modifier mutation through the germline (Figure 1b). While the modifiers enhanced *miniwhite *expression in males indicating that the MSL complex is more active immediately around the *roX1 *transgene, they were unable to stimulate *miniwhite *expression in females (Figure 1d, P864L). The modifiers also overcame the pairing-dependent repression so the change in eye phenotype was more dramatic when the *GMroX1-75C *transgene was homozygous. Mapping and complementation analysis indicated that three mutations are unusual alleles of *msl1 *(Figure 1b, d) and one maps to *mle*, two critical components of the dosage compensation complex.

The enhanced pigmentation we observe with the modifier mutations cannot be due to MSL1 haploinsufficiency, but rather the modifiers must make proteins with altered activities. This was shown by testing the *L60 *deletion [46] in *msl1^L60^*/+; *GMroX1-75C/GMroX1-75C *males and observing no effect on the eye pigmentation pattern (Figure 1d). We recovered essentially equal numbers of *msl1*^Mod^/+ sons compared to either *msl1*^Mod^/+ sisters or +/+ brothers arguing against an antimorphic mechanism (data not shown). Surprisingly, after extraneous secondary mutations were removed by recombination, some homozygous modifier males were recovered which also showed solid red eyes (Figure 1e). The solid red eye phenotype of modifier males argues that mutants have an increased probability of modifying the local chromatin around the *roX1 *insertion. By contrast, the male specific lethality observed in modifier/Df animals demonstrates that the modifiers have reduced ability to dosage compensate the X.

To exclude the possibility that these mutants affected general silencing, we tested their effects on the well characterised position effect variegation (PEV) mutation *In (1) w*^m4^, which displays mosaic pigmentation in both sexes. While *Su(var)2-5 *made the eyes solid red in both sexes as expected [47], our new modifier mutations had no effect (Figure 1f). Furthermore, in mosaic lines where *GMroX1 *transgene is inserted at the telomere of chromosome 2R (60F) or at the 5' end of the *defective proventriculus *(*dve*) gene (58D), each of the three modifier *msl1 *mutations increased red pigmentation in male eyes (Additional file 1) but the females remained pure white. These results show that the effect of the modifiers is completely male specific and largely independent of chromatin context. This indicates that they act on some aspect of the dosage compensation pathway rather than the surrounding repressive chromatin. This is consistent with the finding that most mosaic *GMroX1 *insertions respond weakly or not at all to suppressor of variegation mutations or dose of the heterochromatic Y chromosome [44]. In the specific case of the *GMroX1 *insertion at 75C used for our screen, mutations in *Su(var)2-5*, *Su(var)3-7*, and *su(z)12 *each failed to make the eyes red (data not shown).

### The modifier MSL1 mutations map to the conserved PEHE domain

**Figure 2:**
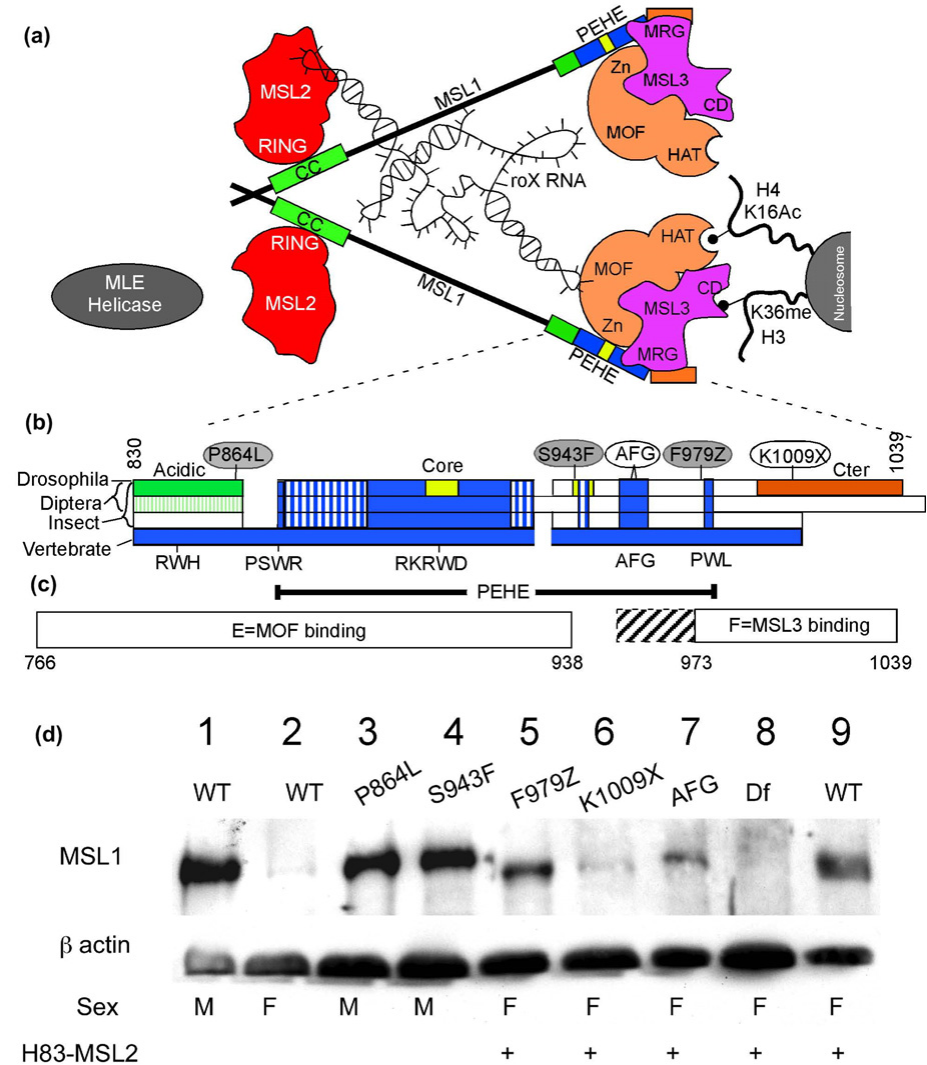
**Male Specific Lethal (MSL)1 modifier mutants map to the conserved C-terminal PEHE domain. (a) **MSL subunit interactions found in previous biochemical and genetic analysis [5,23,25,51,55-57]. CC = coiled coil; HAT = histone acetyltransferase; Zn = Zn finger. Much of the MSL1 sequence is poorly conserved between *Drosophila *species (thin line). MSL1 is portrayed forming dimers at its N-terminus [24]. The chromodomain (CD) of MSL3 has been postulated to bind RNA [58], but here is depicted as binding histone H3 K36me [52,53]. Interactions between RNA and MSL proteins are reported in [26-29,31,58]. **(b) **The sequence alignment presented in Additional file 2 is illustrated graphically. First bar = 12 *Drosophila*. Second bar = three mosquitoes. Third bar = five non-dipteran insects. Fourth bar = 10 vertebrates. When other species share similarity to vertebrates (blue) they are also coloured blue or hatched blue for weak similarity. Open boxes = dissimilar sequences. Three regions are strongly conserved within *Drosophila*, but not found in any other group: a highly acidic region before the PEHE (green), PEHE core domain (yellow), and Cter (orange). Locations of the three modifier alleles (red) and two non-modifier alleles (white) are shown above. The lower labels show the four tryptophan residues and the AFG triplet that serve as sequence landmarks. The PEHE domain (Marin [50]) is shown below. **(c) **Morales *et al. *[25] showed MSL1 fragment E was sufficient to bind MOF ('males absent on first') and fragment F could bind MSL3. We found additional MSL3 contacts occur upstream of codon 979 including the AFG triplet (hatched box). **(d) **Anti-MSL1 western blot. Lane 1, wild-type (wt) male; 2, wt female; 3, *msl1*^P864L ^male; 4, *msl1*^S943F ^male; lanes 5-9, females expressing ectopic MSL2. Females make less MSL1 protein than males [46]; 5, *msl1*^F979Z^; 6, *msl1*^K1009X^; 7, *msl1*^AFG^; 8, *msl1*^L60^; 9, *msl1*^L60^/*CyO*. Loading control, β-actin.

Sequencing showed that the three modifier *msl1 *mutations fell in the most conserved PEHE domain that binds MSL3 and MOF (Figure 2a, b and Additional file 2). Two alleles, P864L and S943F, are missense mutations that changed amino acid residues conserved within the genus *Drosophila*. The third allele, F979Z, is an 11-bp deletion replacing the last 61 amino acids of MSL1 with 25 amino acid residues from another reading frame. These residues are near the previously defined MOF and MSL3 binding sites (Figure 2c). We tested whether simply disrupting the PEHE domain would cause the dominant eye pigmentation effect on mosaic *GMroX1 *transgenes. In previously unpublished screens for *msl1 *mutations, alleles mapping near the C-terminus were recovered that behaved as nulls in male viability assays. The *msl1*^AFG ^allele changes two nearby codons, 965 and 967, from AFG, a triplet conserved from flies to mammals, to EFF (Figure 2b and Additional file 2). The second pre-existing allele, K1009X, generates a stop codon soon after the PEHE domain truncating the last 31 residues. Neither the AFG nor K1009X mutations behaved as modifiers, although AFG had a variable weak effect (Figure 1d and Additional file 1). Thus, the gain of function phenotype seen with P864L, S943F, and F979Z cannot simply be due to disruption of the critical PEHE domain or loss of the fly-specific Cter domain, but instead must alter the activity in some distinctive way. The phenotype and sequences of modifiers suggests that they produce stable MSL1 proteins. Western blot analysis showed this was the case (Figure 2d). Mutant MSL1 can be detected in all samples although K1009X and AFG accumulate to only low levels.

### Complementation between *msl1 *alleles

**Figure 3:**
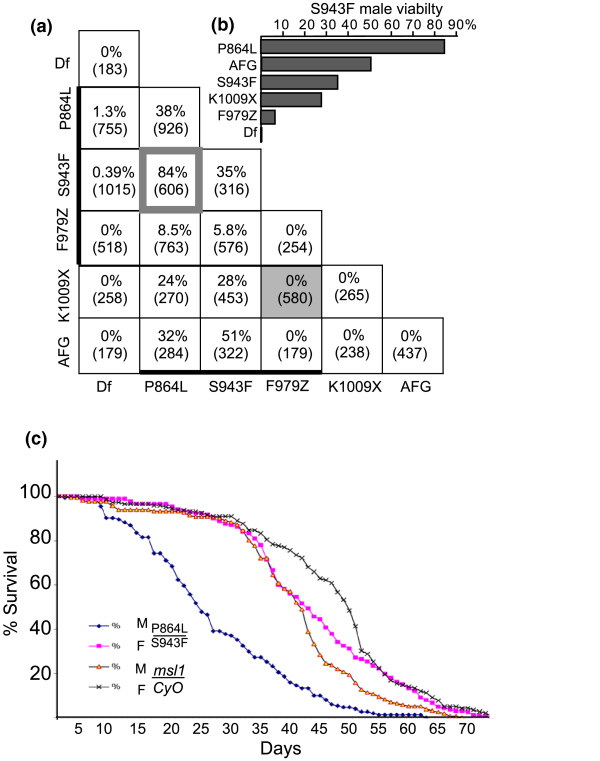
**Interallelic complementation of modifier *msl1 *mutations. (a) **The percentage male viability is shown for each allelic combination. The *msl1 *allele donated by one parent is on the Y axis and the allele donated by the other parent is on the X axis. The Df allele is *msl1^L60^*. The number in parentheses is the number of *msl1*^a^/*msl1*^b ^daughters recovered in each cross. The thicker bars indicate the modifier alleles recovered in the mosaic eye screen. The P864L/S943F heterozygotes used in the longevity assay are boxed. The rare *msl1/Df *male escapers eclosed late, had held out wings, and pale mosaic eyes. **(b) **The viability for all combinations of S943F males are shown with the second allele indicated by the right axis. **(c) **The longevity of the four classes of progeny recovered from a cross between *msl1*^P864L^/*CyO *and *msl1*^S943F^/*CyO *parents. Although *msl1*^P864L^/*msl1*^S943F ^males and females were initially recovered in nearly equal numbers, the adult males appeared weaker, held out their wings, and had shorter lifespans. The starting populations were > 150 adults per genotypic class.

Li and coworkers [24] have presented evidence that MSL1 forms homodimers through a sequence at the extreme N-terminus of the protein. The dominant behaviour of the new modifier mutations might be explained if modifier MSL1 subunits dimerised *in vivo *with wild-type polypeptides made from the other allele. We crossed the three modifier alleles to each other, *AFG*, *K1009X*, and the *msl1^L60 ^*deletion in all possible combinations (Figure 3a). The two missense modifiers are male lethal over a deletion, but produced adult males when homozygous. Thus, these are the first hypomorphic alleles of *msl1 *reported, and demonstrate how exquisitely sensitive flies are to the level of MSL1 activity. Changing the MSL^MOD ^dose by only twofold causes an approximately 100-fold change in male viability normalised to sisters of the same genotype (Figure 3b). All classes of surviving adult males had solid red eye colour (Figure 1d) except rare *mod*/Df escapers that had pale mosaic eyes (data not shown). In addition to strong dose sensitivity, we observed interallelic rescue that is difficult to attribute solely to protein levels. For instance, heterozygous *S943F/AFG *male viability was higher than either homozygote (51% vs 35% and 0%) despite the fact that the AFG protein accumulates to low levels (Figure 2d, lane 7). We can exclude any viability effects caused by secondary mutations along the EMS-treated chromosome, because we measure male viability relative to their homozygous sisters who share any background mutations. Even though some heteroallelic combinations, such as *S943F/P864L*, produced males in nearly Medelian ratios, they had shorter lifespans compared to all other siblings (Figure 3c).

Both *K1009X *and *AFG *were scored as nulls when initially isolated, but their ability to complement *P864L *and *S943F *modifier alleles demonstrates that they retain a subset of functions. In contrast, the P864L and S943F proteins only supported about 7% viability in combination with frameshift modifier, F979Z. This protein is the same length as K1009X but has lost the last few amino acid residues of the PEHE motif followed by scrambled sequence, whereas K1009X carries a wild-type PEHE domain (Figure 2b and Additional file 2). We attribute the greater ability to complement to the presence of a complete PEHE domain in K1009X. These examples of interallelic complementation are most easily accommodated in a model where MSL1 forms dimers within the MSL complex [24]. We saw no complementation between F979Z and K1009X, both of which lack the Cter sequence immediately following PEHE (Figures 2b and 3a).

### Localisation of modifier MSL1 proteins on polytene chromosomes

**Figure 4:**
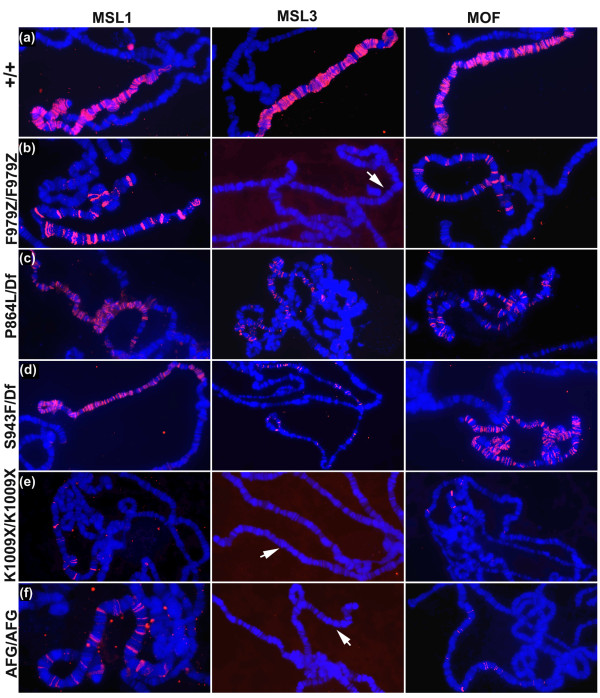
**Modifier *msl1 *mutations have reduced ability to recruit MOF ('males absent on first') and Male Specific Lethal (MSL)3 to the X chromosome *in vivo***. Polytene chromosomes from larvae of the genotype given on the left were treated with antibodies recognising the MSL subunits shown across the top. In all cases blue is DNA and red is the MSL staining. In cases where no MSL binding is evident, an arrow indicates the unstained X chromosome. **(a)**-**(d) **are males, **(e) **and **(f) **are female larvae expressing MSL2.

The pattern of complementation suggested that some mutations might have defects in different functional domains in the protein. To test this idea we first examined whether the mutant MSL1 proteins retained any ability to bind the male X chromosome in the absence of wild-type MSL1. The F979Z frameshift protein bound the X at only about 30-50 bands (Figure 4b). Both P864L/Df and S943F/Df males showed strong painting of the male X despite the fact that these males die before adulthood (Figure 4c, d). The non-modifier AFG and K1009X proteins bound the X very weakly at only approximately 10-25 bands (Figure 4e, f), which could be partially attributed to the lower stability of these proteins (Figure 2d).

We next measured the ability of the mutant MSL1 proteins to recruit the other components of the complex. We found that the MSL2 banding pattern closely resembled the MSL1 pattern in all mutants (data not shown). Both P864L and S943F showed reductions in MSL3 and MOF staining to differing degrees, as might be expected if the amino acid substitutions altered the docking sites for these two subunits (Figure 4c,d). F979Z recruited MOF to the few bands on the X, but no MSL3 was detectable (Figure 4b). The AFG protein weakly recruited MOF, but MSL3 was undetectable even in grossly overexposed photographs (Figure 4f). We also measured *roX1 *RNA levels in these flies. Northern blots showed that the F979Z, K1009X and AFG mutants that fail to recruit MSL3 also had no *roX1 *RNA. By contrast the two missense alleles that painted the X in a nearly normal pattern and could recruit some MSL3 accumulated high levels of *roX1 *(Additional file 3).

### Subunit interactions

**Figure 5:**
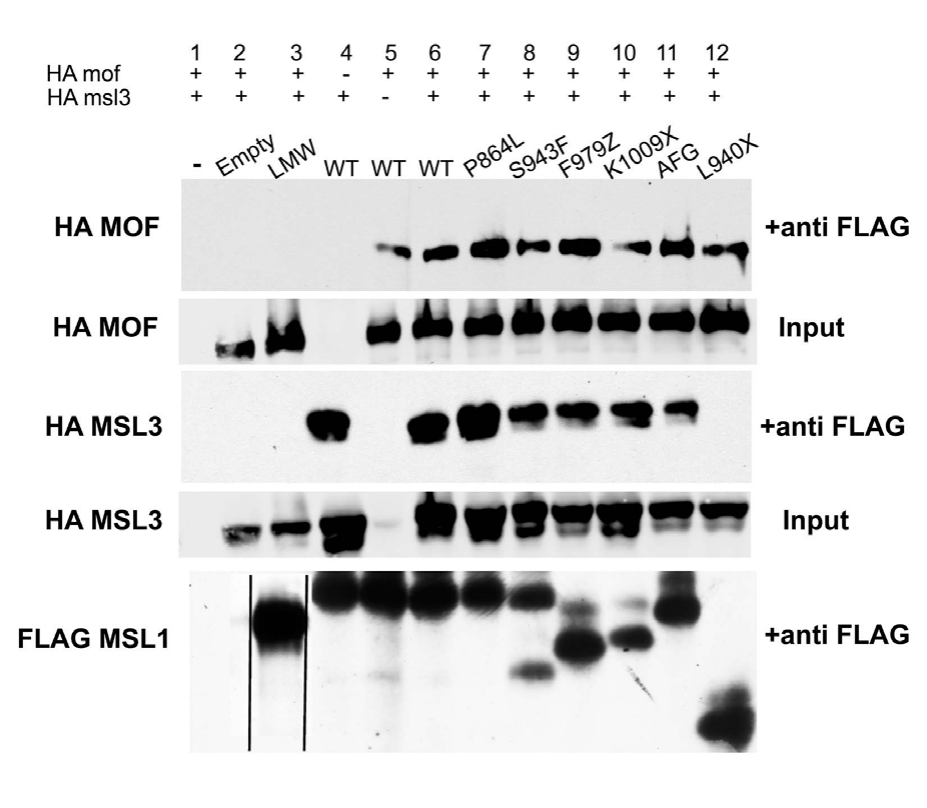
**Mutant Male Specific Lethal (MSL) proteins retain MSL3 and MOF ('males absent on first') binding *in vitro. ***FLAG-tagged MSL1 protein (residues 751-1,039) and either full-length haemagglutinin (HA)-MOF, HA-MSL3, or both were coexpressed in *Drosophila *S2 cells, bound to beads coated with anti-FLAG antibodies, and pelleted. The recovered proteins were visualised with anti-HA antibodies. MSL1 inputs: lane 1, no FLAG plasmid; 2, empty FLAG vector; 3, unrelated FLAG protein of 70 kDa (low molecular weight (LMW)); 4-6, wild-type MSL1; 7, P864L; 8, S943F; 9 F979Z; 10, K1009X; 11, AFG; 12, L940X truncation similar to fragment E in [25]. Bottom: the membrane was stripped and reprobed with antibodies recognising FLAG to visualise the MSL1 proteins. The different sizes reflect different truncations. The unrelated FLAG-tagged protein in lane 2 is much larger than the MSL1 fragments, but was repositioned for comparison.

Polytene chromosome analysis only detects subunit interactions within complexes that were successfully recruited to the X, but any soluble MSL proteins are lost. We used coimmunoprecipitation experiments to test the ability of mutant MSL1 proteins to bind MOF and MSL3 regardless of whether they also bound the X. FLAG-tagged MSL1 fragments encompassing residues 751-1,039 were coexpressed in S2 cells with either full length MSL3 or full length MOF each tagged with haemagglutinin (HA) (Figure 5 and summarised in Additional file 4). All five MSL1 mutant proteins readily interacted with both MSL3 and MOF, although the missense mutants S943F and P864L were slightly stronger and AFG showed somewhat reduced binding to MSL3. The ability of most MSL1 mutants to bind both MSL3 and MOF was unexpected given the results with polytene chromosomes. In particular, the two C-terminal truncations, K1009X and F979Z, each bound MOF weakly and MSL3 not at all on the polytene chromosomes (Figure 4), but precipitated MSL3 and MOF efficiently from S2 cells (Figure 5, lanes 9 and 10). This indicates that the PEHE domain is sufficient for MSL3 interaction in solution, but that the Cter domain is essential to stably tether MSL3 to the X chromosome. Previous work reported that the Cter rather than the PEHE domain was sufficient for strong MSL1-MSL3 binding (Morales *et al. *[25]). We agree that critical MSL1-MSL3 interactions map to the Cter, but consider the newly observed PEHE-MSL3 interactions to be authentic for three reasons. First, they were obtained after 1 M NaCl washes. Second, an unrelated FLAG-tagged protein (Figure 5, low molecular weight (LMW) lane 2) was unable to precipitate either MOF or MSL3. Finally, we assayed a truncation encompassing MSL1 residues 766-939, L940X, equivalent to fragment F assayed by Morales *et al. *[25]. L940X precipitated MOF but not MSL3 as described earlier (Morales *et al. *[25] and Figure 5 lane 12) demonstrating that our assay conditions are comparable to those used by others. These observations argue against non-physiological binding between MSL3 and K1009X or F979Z due to over expression in cell culture, and instead, suggest that MSL1 binds MSL3 by two distinct domains (Additional file 4).

### MSL1 mutants abolish MSL complex cis spreading

**Figure 6:**
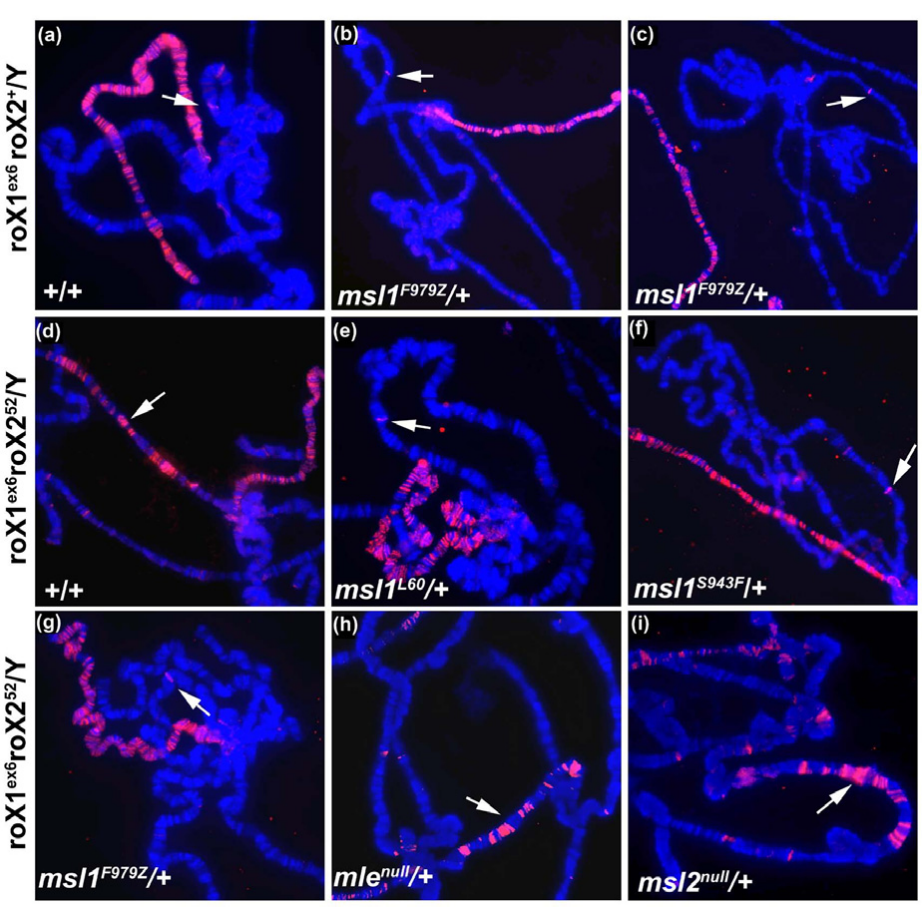
**Mutations in *msl1 *abolish local Male Specific Lethal (MSL) spreading**. In all cases except **(c)**, polytene chromosomes were taken from males carrying one copy of *GMroX1-75C *inserted on the third chromosome (arrow). In **(c) **the transgene is homozygous. Chromosomes were stained with anti-MSL1 antibodies (red). In **(a)**-**(c) **the X chromosome is *roX1*^ex6 ^*roX2*^+^. **(a) ***msl1*^+^/*msl1*^+^, **(b) **and **(c) ***msl1*^F979Z^/*msl1*^+^. In **(d)**-**(i) **the X chromosome is *roX1*^ex6 ^*roX2*^52^. **(d) ***msl1*^+^/*msl1*^+ ^the MSL complex covers 73A-77C, **(e) ***msl1*^+^/*msl1*^L60^, **(f) ***msl1*^+^/*msl1*^K1009X^, **(g) ***msl1*^+^/*msl1^F979Z^*, P864L, AFG and K1009X also fail to support local MSL spreading (not shown), **(h) ***mle*^+^/*mle*^null^, the mle^I926Z ^allele was also examined and gave identical results (not shown), **(i) ***msl2*^+^/*msl2*^null^. Dense MSL1 staining occurs over the X chromosome.

When MSL complex contains RNA derived from an autosomal *roX1 *transgene, it can either diffuse to the X *in trans*, or spread *in cis *around the transgene. This choice is strongly affected by the amount of MSL subunits available for assembly, the rate of *roX1 *transcription, and the presence of any other source of competing *roX *transcripts [30,32]. We tested whether the red eye phenotype seen in our MSL1 modifier mutants was due to enhanced *cis *spreading from *roX1 *transgenes, as observed in *roX1 roX2 *mutants (Figure 1a). No local autosomal MSL spreading was seen in *roX1*^- ^*roX2*^+^/Y; F979Z/+ males regardless whether the *GMroX1-75C *transgene was hemizygous or homozygous (Figure 6b,c). Only a single MSL1 band at the 75C insertion site was detectable, and this is due to binding to a well characterised internal enhancer element [48]. The other two modifier alleles did not support widespread spreading either (data not shown). This shows that the red eye colour phenotype of modifier MSL1 mutants is not the result of enhanced long distance MSL spreading around the *GMroX1 *transgene.

We next examined the MSL complex distribution in males whose only source of *roX *RNA was the *GMroX1-75C *transgene. While robust autosomal MSL spreading (> 1 Mbp) consistently occurred around the *GMroX1-75C *transgene in nearly all nuclei of *msl1*^+ ^homozygotes (Figure 6d), spreading was abolished in males heterozygous for any of the five *msl1 *mutations or deletion (Figure 6e,f). Not only do the modifiers not enhance local spreading, instead they totally block spreading even under conditions where it is normally favoured. We went on to test whether mutations in any MSL subunit would likewise dominantly abolish local spreading. We found that *msl2*^1^/+, *mle*^1^/+ and *mle^I926Z/+ ^*heterozygous males continued to support massive *cis *spreading from *GMroX1-75C *(Figure 6h,i). This indicates that *cis *spreading is particularly dependent upon MSL1.

### Modifier MSL1 proteins support dosage compensation using autosomal *roX1 *RNA

Finding that *msl1 *mutations abolished *cis *spreading of mature MSL complexes around autosomal *roX1 *transgenes despite the presence of wild-type MSL1 subunits, we asked if these same flies had impaired ability to dosage compensate the X chromosome *in trans*. We measured the ability of the different *msl1 *mutants to support dosage compensation when the only source of *roX *RNA was the autosomal transgene. Males that lack the endogenous *roX1 *and *roX2 *genes die, but can be partially rescued by an autosomal *GMroX1 *transgenes [32,49]. Northern blots showed that *roX1 *RNA levels produced by *GMroX1-75C *are comparable to wild-type levels (data not shown). We found that an autosomal *GMroX1 *transgene could not restore male viability if the amount of MSL1 was reduced by half in *msl1*^L60^/+ hemizygous males (Table 1). This assay clearly divided the *msl1 *point mutants into distinct classes. The three modifier alleles that enhance eye pigmentation in *GMroX1*-*75C *mosaic males restored male viability when heterozygous with one copy of *msl1*^+ ^(Table 1). The *AFG *and *K1009X *non-modifier alleles behaved more like simple loss of function *L60 *deletion in this assay. The ability of the modifier alleles to restore dosage compensation to the X nearly as well as wild-type MSL1 demonstrates that such complexes have essentially normal chromatin remodelling activity, despite having dramatically impaired ability to spread locally.

**Table 1:** Modifier alleles of *msl1 *retain much of their activity

*msl1 *allele	*msl1*/+ male viability (%)	N
+	51	397

L60	3.4	180

P864L	42	239

S943F	32	191

F979Z	30	288

AFG	8.8	193

K1009X	6.0	247

The viability of *roX1 roX2*/Y; *msl1*/+; [*w^+ ^GMroX1-75C*]/+ males was measured when the only source of *roX *RNA was the autosomal transgene. In each case the progeny were from *roX1 roX2 *mothers mated to *roX*^+^/Y; *msl1*/*CyO*; [*w^+ ^GMroX1-75C*]/[*w^+ ^GMroX1-75C*] fathers. The *CyO *progeny were discarded and the fraction of *msl1*/+ sons and daughters was compared. The first column indicates which *msl1 *allele was used. The middle column shows the percentage male viability compared to their sisters, and the right column gives the number of *msl1*/+ females recovered in each cross.

### Modifier mutation in the MLE RNA helicase

**Figure 7:**
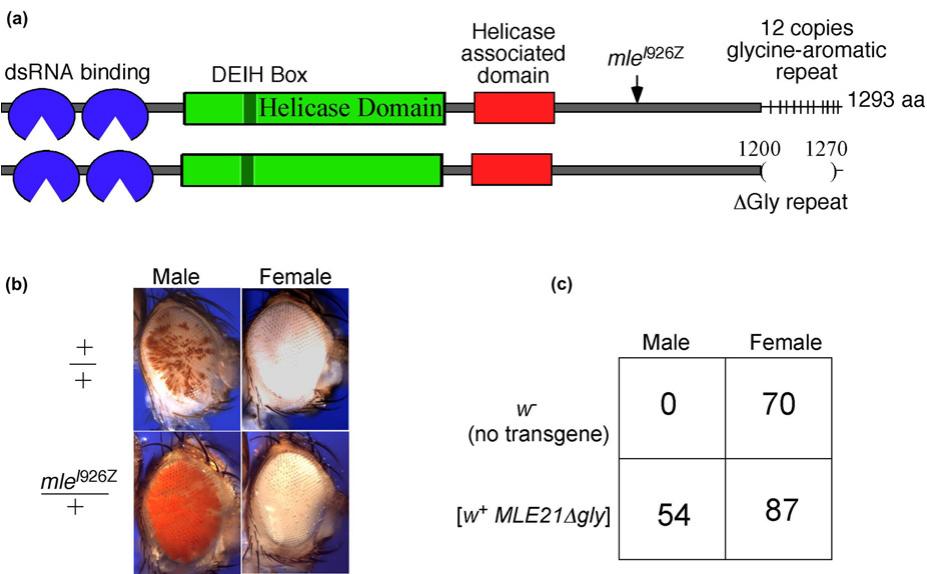
**A late truncation in MLE (for 'maleless', RNA helicase) produces a modifier phenotype**. **(a) **The MLE protein contains two double stranded RNA binding motifs at the N-terminus and a large helicase domain in the middle. The C-terminus has 12 imperfect copies of a glycine-rich heptad repeat, each of which has 1 aromatic residue. The modifier allele has a 1-bp deletion in the I926 codon. The *MLE21Δgly *transgene carries a deletion of the heptad repeats on a genomic clone expressed from its native promoter. **(b) **Male and female homozygous for [*w*^+ ^*GMroX1-75C*] who are either *mle*^+^/CyO (top) or *mle*^I926Z^/CyO (bottom). **(c) **The *MLE21Δgly *transgene rescues male viability, but not fertility. *y w; mle*^1^/*CyO y*^+^; [*w*^+ ^*MLE21Δgly*]/+ males and females were mated and the homozygous *mle*^1^/*mle*^1 ^progeny were counted and scored for eye colour and sex. The *MLE21Δgly *construct could not be assayed for its effect on mosaic eyes of *GMroX1-75C *because of the linked *w*^+ ^marker.

The weakest modifier recovered in our screen was a late frameshift mutation in the MLE RNA helicase subunit of the MSL complex (Figure 7a, b). As was the case with *msl1*, null mutations in *mle *do not act as modifiers (data not shown). The *mle^I926Z ^*allele displays a male-specific lethal phenotype when homozygous or in combination with null alleles of *mle*. This mutation does not affect long distance MSL spreading from sites of *roX1 *transcription (data not shown). Because the mutant protein retains the two N-terminal double stranded (ds)RNA binding motifs and helicase region, we asked if the recessive male lethality might be due to loss of the distinct glycine-aromatic heptad repeats at the C-terminus of MLE, postulated to bind RNA [27]. We constructed an *mle *transgene lacking the repeats (Figure 7a) but found that it supported male viability (Figure 7c), but the rescued males were sterile. This shows that the heptad repeat is needed for its germline function, but not for dosage compensation. Thus, male lethality displayed by the modifier allele is most likely due to the loss of some element required for dosage compensation located between codons 925 and 1,200.

## Discussion

Genetic screens assaying for male-specific lethality identified the known components of the dosage compensation complex, and yet the precise mechanism by which the MSL complex acts upon RNA polymerase is not understood. We developed a new approach to search for factors that are needed for either the activity or targeting of the MSL complex using a simple eye pigmentation assay. This dominant screen has the potential to recover genes that would have been overlooked previously because they perform additional essential functions in females. In fact, two of the new mutants are recessive lethal to females as well as males. Based on the following criteria we consider the mutants isolated by this strategy relevant to the process of dosage compensation. (1) All modifiers alter eye pigmentation exclusively in males. (2) The modifiers have no effect on general position effect variegation. (3) The modifiers act on *roX1 *transgene inserted in diverse repressive environments indicating that they act on *roX1 *rather than relieving flanking repressive chromatin. (4) Of the 13 mutants recovered, 4 mapped to genes encoding known components of MSL dosage compensation complex. Here we focused on the unusual characteristics of the new *msl1 *mutations because their eye colour phenotype indicates increased dosage compensation, yet in most assays they behave as loss of function.

A chromatin-remodelling machine similar to the MSL complex can be found in most animals where it probably acetylates histone H4 at many genes in both sexes. Within the genus *Drosophila *this complex has been recruited to X-linked genes in males to carry out dosage compensation. This specialisation almost certainly required changes in one or more of the MSL subunits allowing them to interact with *roX *RNAs and bind only the male X. The most conserved region of MSL1 proteins from insects to vertebrates is termed the PEHE motif [50]. The PEHE domain was known to bind the MOF histone acetyltransferase [23,25]. A short interval in the middle of the PEHE, which we term the Core (yellow box in Figure 2b and Additional file 2), is strongly conserved among most insects and vertebrates, but is significantly different in *Drosophila*. Immediately upstream of the PEHE domain is a highly acidic region and downstream is a second strongly conserved block, Cter, found only within the genus *Drosophila *(Figure 2b). The acidic motif, Core, and Cter *Drosophila*-specific domains are candidate adaptations needed for MSL complex to carry out the new role of dosage compensation. Our mutational analysis highlights the roles of PEHE and Cter.

The three modifier mutations recovered in *msl1 *cluster around the PEHE domain. The two missense mutants display an unusual constellation of phenotypes. When heterozygous with a wild-type allele, the males are fully viable showing that the X is compensated, but more importantly, the eye colour shifts from mosaic to solid red. This shows that MSL complexes containing the mutant protein are more effective at modifying chromatin immediately around sites of *roX1 *transcription so that the adjacent *miniwhite *marker has a high probability of being epigenetically activated. The same mutations are male specific lethal over a deletion indicating loss of function. While these proteins paint the X in a nearly normal pattern, they can only partially recruit MOF and MSL3 to the X. MSL complexes lacking MSL3 are restricted to approximately 35 high affinity sites along the X [51], so finding a high density of MSL1 bands in the modifiers suggests that MSL3 likely associates transiently with mutant MSL1 at all sites *in vivo *even if it is difficult to capture with our fixation conditions. This is in agreement with our finding that the modifier MSL1 mutants bind MSL3 efficiently in precipitation assays (Figure 5).

The Cter domain (residues 994-1,039) is strongly conserved within the genus but shows no resemblance to the C-terminus in mosquitoes or other insects (Additional file 2). This domain is essential for dosage compensation *in vivo *as shown by the male-specific lethal phenotype of the late nonsense mutant K1009X. The initial mapping of MSL1-MSL3 interactions to the Cter domain [25] was surprising because this domain is not conserved outside *Drosophila*, and yet MSL1 binds MSL3 even in vertebrates. Here we resolve this issue by showing that the conserved PEHE domain carries a second MSL3 binding region. While our observations that K1009X and F979Z fail to recruit MSL3 to polytene chromosomes is fully in agreement with earlier work, we were initially surprised to find both truncations robustly precipitated MSL3 in coimmunoprecipitation experiments in cell culture. The ability of K1009X and F979Z to bind MSL3 maps to the second half of the PEHE domain (Figure 2c). This is consistent with the observation that mutations in the highly conserved AFG residues within the PEHE domain abolish MSL3 binding *in vivo *despite the presence of the Cter domain. This argues that the PEHE domain makes critical contacts with both MSL3 and MOF that are likely to be evolutionarily ancient because only the PEHE domain is conserved through vertebrates. The new mosaic eye assay used in this screen apparently selected for mutants that subtly altered subunit interactions without completely abolishing them. We propose that the Cter motif exclusive to *Drosophila *provides additional contacts necessary for the recently acquired function of dosage compensation. We note that removing this C-terminal domain results in loss of *roX1 *RNA (Additional file 3) as expected if this motif plays some new role in aiding MSL3 target the X in conjunction with *roX *RNAs.

We propose that the PEHE and Cter domains have distinct functions based on the observation that the missense modifier mutants restore partial male viability to K1009X and AFG mutants, but not to an *msl1 *deletion. If each mutant affected distinct functions within MSL1, then males making mixed MSL complexes might partially restore function. However, the F979Z modifier only weakly complements P864L and S943F. Although both F979Z and K1009X delete the Cter domain, F979Z also deletes the last few conserved residues of the PEHE domain explaining the reduced ability to complement. This would also explain why F979Z fails to complement AFG. These complex complementation patterns are most easily explained if MSL1 dimerises within MSL complexes [24].

Prior to this study, the only factor affecting the mosaic *miniwhite *expression linked to *roX1 *transgenes was removing the endogenous *roX1 *and *roX2 *genes (Park *et al*, [32]). This also results in massive local spreading of the MSL complex around the *roX *transgene. We were therefore surprised to find that while the dominant *msl1 *modifiers have the same affect on the mosaic eye pattern as *roX *deletions, the modifiers abolished *cis *spreading rather than enhancing it. Massive local spreading appears to be coupled to cotranscriptional assembly of MSL subunits onto nascent *roX *transcripts [30,31]. Under conditions where assembly is completed by the time the 3' end of *roX1 *RNA is released from polymerase, local spreading is favoured. When partially completed *roX *transcripts are released before assembly is completed, the inactive complex diffuses away from the site of *roX *synthesis preventing local spreading. The *msl1 *mutants characterised here block local spreading in a dominant fashion even when wild-type subunits are available. We propose this is a direct reflection of how the mutant MSL1 subunits affect the assembly process.

Flies are exquisitely sensitive to the dose of MSL1. When MSL2 is ectopically expressed in females, only about 15% of the animals reach adulthood and are sterile. Simply reducing MSL1 by half completely relieves this toxicity [12]. However, overexpressing MSL1 in the presence of ectopic MSL2 kills 100% of females [46]. In the present study we found that the viability of males carrying modifier mutations P864L or S943F changes drastically over only a twofold change in protein levels. We also found that reducing the MSL1 dose by half in *msl1*^L60^/+ males is sufficient to block distant *cis *spreading of the MSL complex. This suggests that the availability of MSL1 subunits is the most limiting factor controlling assembly onto *roX *RNAs. Although MSL1-MSL2 heterodimers are thought to form the core of the dosage compensation complex, we propose MSL1 has a stronger effect on assembly kinetics because it alone recruits MSL3 and MOF to the complex. The modifier alleles isolated here reinforce this model. In this case they act not by altering the abundance of MSL1, but rather act at the MSL1:MOF and MSL1:MSL3 interfaces. The two missense MSL1 modifier proteins are produced in amounts comparable to wild type (Figure 2d), stabilise *roX1 *RNA (Additional file 3), are incorporated into MSL complexes and paint the X in a nearly normal pattern (Figure 4). Their capacity to block mixed complexes containing wild-type MSL1 from spreading is more likely due to problems coupling MSL assembly with 3' processing and release of *roX1 *transcripts. The red eye phenotype indicates that complexes tend to accumulate immediately around the site of *roX1 *transcription, activating *miniwhite*. The role of MSL3 in spreading may be its ability to recognise actively transcribed genes by their histone H3K36me3 mark [52,53]. This might explain why alterations around the PEHE domain that affect whether or how MSL3 is presented to chromatin would inhibit extensive *cis *spreading.

We also isolated a single modifier allele in *mle *that is a late frame shift that retains the two dsRNA binding motifs and the large helicase domain. As in the case of the *msl1 *modifier alleles, the *mle *modifier displays recessive male lethality in addition to the dominant effect on mosaic eye colour. We showed that the male lethality cannot be attributed to the aromatic repeat sequence at the C-terminus, but rather must be located more upstream in a poorly characterised region of the protein. The biochemical role of MLE in dosage compensation is not known, but it is tempting to speculate that it acts on *roX *RNAs, either during assembly of the MSL complex, or to catalyse conformational changes in the complex either during cycles of histone modification, or movement along the chromosome.

## Conclusions

We developed a new genetic strategy to identify components of the dosage compensation pathway in *Drosophila *using a simple dominant eye colour phenotype. This yielded new mutation of *msl1 *and *mle *with novel phenotypes never observed in previously isolated alleles, which are likely due to disruption in specific subunit contacts that alter complex assembly. We also recovered mutations in new genes not previously linked to dosage compensation that have additional functions in females.

## Methods

### Fly stocks

The *GMroX1-75C *transgenic stock was as previously described [44]. The *roX1 *transgenes located at 60F and 58D contain a 1-kb insertion of foreign sequence at the extreme 3' end between roxbox2 and roxbox3. This insertion lowers, but does not eliminate activity. The *msl1 *alleles *L60*, *AFG*, and *K1009X *(along with 30 others) were isolated previously in Mitzi Kuroda's laboratory (Harvard-Partners Ctr. for Genetics and Genomics, Harvard Medical School, 77 Avenue Louis Pasteur, NRB 168, Boston, MA 02115, USA) during a γ-ray mutagenesis selecting for females that could tolerate ectopic MSL2 expression similar to that reported in Lyman *et al. *[51]. The full genotype of the *roX1 rox2 *double mutant stock is *y w roX1*^ex6 ^*Df(1) roX2*^52 ^*nod *[*w*^+^*cos4Δ*] [54]. For eye colour assays we use a stock containing a *w*^- ^derivative of [*cos4Δ*] located on the second chromosome (a gift from Victoria Meller, Department of Biological Sciences, Wayne State University, 5047 Gullen Mall, Detroit, MI 48202, USA).

### Mutagenesis

Males isogenic for the second chromosome were fed 25 mM EMS overnight, rested 1 day and then mated to *GMroX1-75C *virgins. The modifier candidate males recovered initially showed an extremely weak phenotype, but males in later generations developed a dramatically darker red eye phenotype after brother-sister matings resulted in progeny homozygous for the *GMroX1-75C *transgene. The design of the screen prevented recovery of X-linked mutations. Third chromosome mutations were unlikely to be recovered because we later learned that the eye phenotype required animals to be homozygous for the unmutagenised third chromosome carrying *GMroX1-75C*. Several stocks initially carried extraneous secondary recessive lethal mutations that were later removed by recombination.

### Complementation tests

Balanced males from each of the 13 modifier mutants were mated to *w*; *msl1*^L60^/*CyO*, *w*; *msl2*^1^/*CyO*, or *w*; *mle*^1^/*CyO *virgins, and the presence or absence of flat wing males was scored. Interallelic complementation tests were performed between *msl1*/CyO mutations carrying [*w^+ ^GMroX1-75C*], with partial rescue measured as the number of adult heteroallelic sons recovered compared to sisters of the same genotype. Lifespan assays were performed on freshly eclosed adults housed 30 animals of the same genotype and sex per vial on standard molasses food without yeast supplement. The survivors were counted on alternate days and then placed in fresh food vials.

### Sequencing

The genomic DNA from either homozygous or hemizygous adult females was recovered, amplified with sets of gene-specific primers pairs, and then sequenced from each end of the fragment. The sequence of each mutation is given in Additional file 3.

Sequencing primers for *msl1 *were: primer 1R 5' GGAGACTCCTTCATGTTGATACC 3'; primer 1L 5' GAATTATGAGATCGTAGGACCG 3'; primer 2R 5' CGCTTCTAATGCATCTACCAT 3'; primer 2L 5' CACACAAACGATAGATGCG 3'; primer 3R 5' CGTAACCTGTGACGAATGAC 3'; primer 3L 5' GGAAATCAGAATCGGATAACT 3'; primer 4R 5' TGAACTGTCACCTCGTTGA 3'; primer 4L 5' GTCTCAGAGCCCAGATCAAG 3'; primer 5R 5' GTTAACTCTGGTGCTTTCACGTT 3'; primer 5L 5' GAAGGAGCAGATACGGCTT 3'.

Primer pairs used to sequence *mle*^I926Z ^were: mleP1L 5' TTAATCGATATCAGAATAGAC 3'; mleP1R 5' CATCGTGGTTTAGAGGGCGATAGG 3'; mleP2L 5' CAGCGGATGCTGGTGCTTCGG 3'; mleP2R 5'GATTTGGCCGTAGGCCATATTC 3'; mleP3L 5' GACTCCCACGATAGCCCGAGG 3'; mleP3R 5'ATCTCTAAACTGTTTTTTAATAC 3'; mleP4L 5' TCTGTTATCTCTTACATTCATACCC 3'; mleP4R 5'AGTGTCGCCCAATTGCTCGCACCGC 3'; mleP5L 5' ACCCAGATTGCCCAATACATTCTT 3'; mleP5R 5'AATTGTCGAAGAGTTTCACCCC 3'; mleP6L 5' GTCATACATTTCTAGTCCTAATTT 3'; mleP6R 5'TTCTGGAACAGGCTCGAAAACCTTGCGT 3'; mleP7L 5' AACCTGATCTTTGCGCTTATGAAGT 3'; mleP7R 5'CCGAGGACCATCATCTTTCCAAGT 3'; mleP8L 5' GGAGCCACCTCCGGTAGACGCAGT 3'; mleP8R 5'CCAAGTTGCTGCAATTCACCGAGG 3'; mleP9L 5' GGGATGACCCCGTGCTGGATGTG 3'; mleP9R 5'ATTATTTCCATATCCTCCTCCA 3'; mleP10L 5' GAATCAGGAATTCTGCCGCACCAATC 3'; mleP10R 5'CACATGCGTATTTAATGCCAAAAA 3'.

### Deleting MLE glycine repeat

The region immediately upstream of the glycine repeats was amplified with MLER1 5' GCAGCGTGAATCAG**GAATTC **3'; ASCBGL 5' CGCTTTACTAACAGCAGCTTGGCGCGCCGTGGAAACGGCAGAT 3'. The region immediately downstream of the glycine repeats was amplified with: LGBCSA 5' **CGCC**GTGGAAACGGC**AGATCT**TTTGGAGGAGGATATGG 3'; BSNOT 5' GTTACCCCA**GCGGCCGC**C 3'. The two resulting PCR fragments were joined by PCR with the two outside primers MLER1 and BSNOT. This fragment lacking the glycine repeats was substituted for the wild-type sequence in an MLE21 backbone [27].

### Polytene chromosome staining

This was performed as previously described [51].

### Northern blots

Total RNA from either sexed adults or wandering third instar larvae was extracted with Trizol reagent (Invitrogen: http://www.invitrogen.com) as directed by the supplier and 10 μg was run on 1.0% agarose in 3-(*N*-morpholino)propanesulfonic acid (MOPS) formaldehyde gels. The RNA was transferred to Hybond N+ membranes in 50 mM NaOH and then hybridised to dsDNA probes labelled by random priming.

### Recombinant DNA

To make the FLAG-tagged MSL1 proteins, genomic DNA was prepared from homozygous F979Z, K1009X and AFG females, hemizygous S943F and P864L males and wild-type males. The last 870 bp of the *msl1 *gene encoding amino acids 751-1,039 was amplified using the following primers: MSL1CTF 5' ACA**AGATCT**CCACACCAACGCCTGGCTCA 3'; MSL1CTR 5' CCA**CTCGAG**CTAACGATTCTTCTGGCGCTT 3'.

The primers contained either *Bgl*II or *Xho*I sites (bold). FLAG MSL1CT constructs were made by digesting the appropriate PCR fragments by *Bgl*II and *Xho*I, then cloning into *Bgl*II *Xho*I double digested pUAST FLAG vector. The *msl1 *L940X mutation was made with the primer 5' CAAAACTCGAGCTAAGGATCCAGCGCAACCAAC 3' instead of MSL1CTR. The cloning of all constructs was confirmed by sequencing. The FLAG MSL1CT expression was driven by cotransfecting p*Actin:GAL4 *plasmid (a gift from Hugo Bellen, Department of Molecular and Human Genetics, HHMI, Baylor College of Medicine, One Baylor Plaza, Houston, TX 77030, USA).

pCasper.HA mof and pCasper.HA msl3 were kind gifts from Max Scott (Department of Genetics, North Carolina State University, Campus Box 7614, Raleigh, NC 27695-7614, USA) [23]. The HA.mof and HA.msl3 inserts were subcloned into pET28a vector between *Eco*RI and *Sal*I sites. pET.HAmof was digested with *Eco*RI and *Xho*I, the HAmof fragment was gel purified, and cloned into *Eco*RI *Xho*I digested pAC5.1/V5 His-A vector. pET.HAmsl3 was digested with *Eco*RI and *Not*I, the HAmsl3 insert was gel purified, and cloned into *Eco*RI and *Not*I digested pAc5.1/V5 His-A vector.

### Coimmunoprecipitation

S2 cells were plated at a density of 1 × 10^6 ^cells/ml in six-well plates. After 24 h the cells were transfected with 400 ng of total DNA mixture of equal proportions of the appropriate plasmids using the Effectene transfection reagent (Qiagen Inc, 27220 Turnberry Lane, Valencia, CA 91355, USA). After 36 h the cells were harvested and washed twice with ice-cold phosphate-buffered saline (PBS). Cells were pelleted and resuspended in 200 μl of lysis buffer (50 mM tris(hydroxymethyl)aminomethane (Tris)-HCl (pH 8.0), 100 mM NaCl, 1% nonyl phenoxylpolyethoxylethanol (NP40), 10% glycerol, 1.5 mM ethylenediaminetetraacetic acid (EDTA; pH 8.0) and protease inhibitor (Sigma-Aldrich, Customer Service, PO Box 14508, St. Louis, MO 63178, USA). Lysis was performed by rotating in lysis buffer at 4°C for 30 min. The lysate was cleared by spinning at 13,000 rpm at 4°C for 10 min. EZ view FLAGM2 beads from Sigma were equilibrated in the lysis buffer and 30 μl of 50% bead slurry was used for each precipitation. A total of 180 μl of the lysate and 30 μl of the 50% bead slurry was rotated overnight at 4°C. The beads were pelleted by spinning at 2,500 rpm for 30 s and washed thrice with washing buffer (50 mM Tris-HCl (pH 8.0), 1 M NaCl, 1% NP40, 10% glycerol, 1.5 mM EDTA (pH 8.0) and protease inhibitor (Sigma)). After a final wash in low salt (150 mM) buffer, the beads were suspended in 20 μl of low salt wash buffer and SDS loading dye.

### Western blots

The entire sample was boiled with SDS loading dye for 3 min and loaded onto an 8% SDS-polyacrylamide gel. Proteins were transferred in Tris-glycine buffer (pH 8.3) containing 25 mM Tris and 192 mM glycine and 20% methanol, for 1.5 h at 400 mA. Membranes were incubated with anti-HA antibody (Covance Research Products, Inc., 5858 Horton Street, Suite 500, Emeryville, CA 94608, USA) overnight at 4°C. After three 15-min washes in PBS-Tween and 2 h incubation at room temperature with anti-mouse horseradish peroxidase conjugate antibody (Jackson ImmunoResearch Laborotories, Inc., PO Box 9, 872 West Baltimore Pike, West Grove, PA, 19390, USA), the proteins were detected with luminol system (Santa Cruz Biotechnology, Inc., 2145 Delaware Avenue, Santa Cruz, CA 95060, USA).

## Competing interests

The authors declare they have no competing interests.

## Authors' contributions

MP performed most of the experiments. RLK conceived the project, designed most of the experiments and performed a few of them. Both authors wrote the paper together.

## Supplementary Material

Additional file 1 **Figure S1**. *msl1 *modifiers act on *GMroX1 *transgenes located at different repressive chromatin environments. Click here for file

Additional file 2 **Figure S2**. Sequence alignment of Male Specific Lethal (MSL)1 C-terminus. Click here for file

Additional file 3 **Figure S3**. *roX1 *accumulation in *msl1 *mutants. Click here for file

Additional file 4 **Figure S4**. Male Specific Lethal (MSL)1 binding proteins. Click here for file
