# Supplementary material for: A new strategy for isolating genes controlling dosage compensation in Drosophila using a simple epigenetic mosaic eye phenotype
Source: BMC Biol. 2010 Jun 10;8:80. doi: 10.1186/1741-7007-8-80 (PMC2893135; doi:10.1186/1741-7007-8-80)
Supplement: Additional file 1 — Figure S1. msl1 modifiers act on GMroX1 transgenes located at different repressive chromatin environments. [file 1741-7007-8-80-S1.DOC]

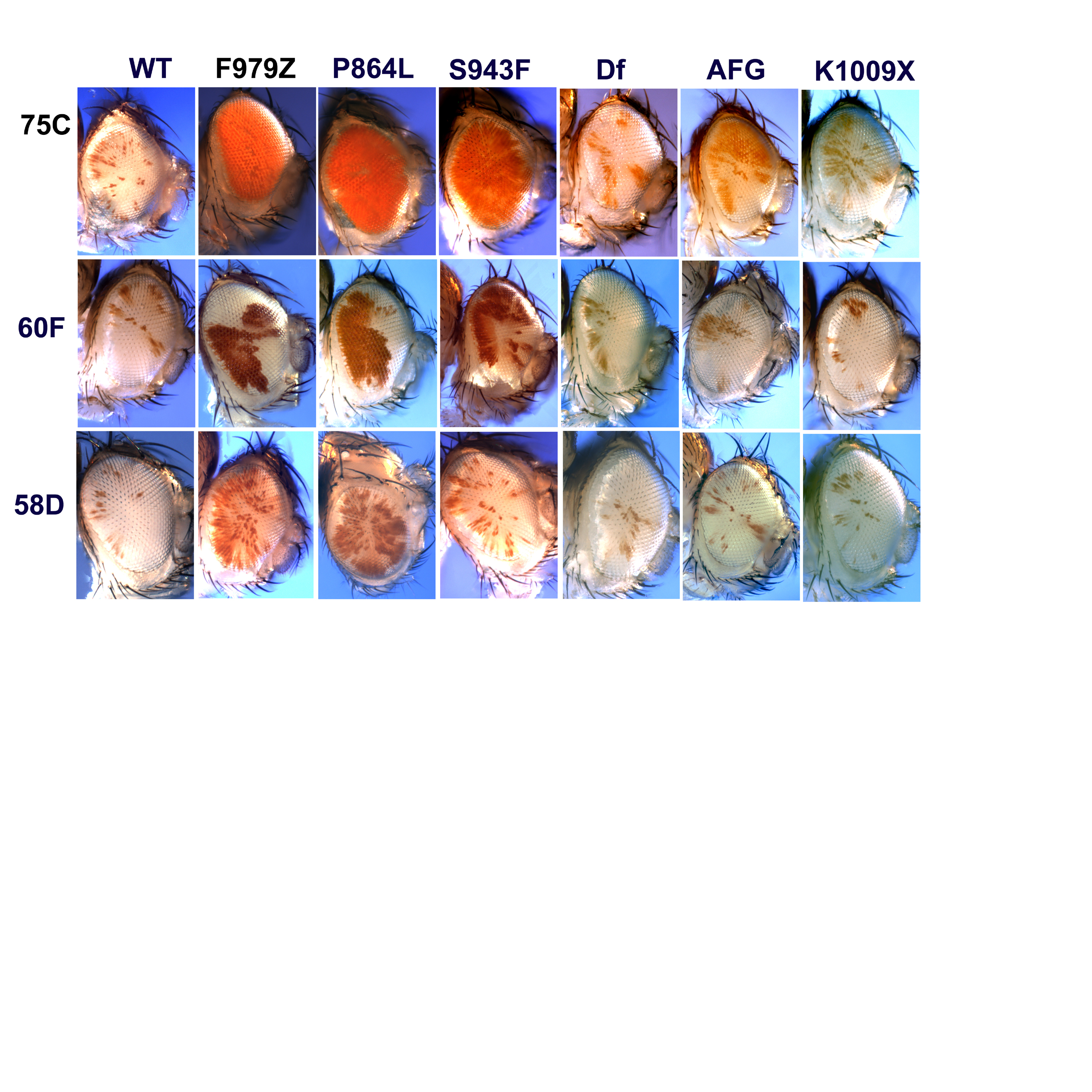


Figure S1. *msl1* modifiers act on *GMroX1* transgenes located at different repressive chromatin environments. All flies carry one wild type allele of *msl1* and the other allele is indicated across the top row. Each row shows males homozygous for a *GMroX1* inserted in different repressive environments. 75C in a middle repetitive YOYO element, 60F in telomeric DNA, and 58D at the 5’ end of an ordinary euchromatic gene, *dve*. Males display mosaic *miniwhite* expression and the females have pure white eyes (not shown). Mutants F979Z, P864L and S943F were identified in the current screen and act as dominant enhancers of mosaic *miniwhite* expression on *GMroX1* insertions at 75C and 60F. Mutants F979Z and P864L also act at 58D. L60 is a nearly complete deletion of the *msl1* gene (Chang and Kuroda 1998) and has no effect on the mosaic pattern. AFG and K1009X are previously identified lesions that map near the modifier mutations, but have little or no effect on the mosaic *miniwhite* expression. None of the *msl1* mutants have any effect on *miniwhite* expression in females (Fig. 1D and data not shown).
