# Supplementary material for: A new strategy for isolating genes controlling dosage compensation in Drosophila using a simple epigenetic mosaic eye phenotype
Source: BMC Biol. 2010 Jun 10;8:80. doi: 10.1186/1741-7007-8-80 (PMC2893135; doi:10.1186/1741-7007-8-80)
Supplement: Additional file 2 — Figure S2. Sequence alignment of Male Specific Lethal (MSL)1 C-terminus. [file 1741-7007-8-80-S2.DOC]

A

| --------A D N P S L E  WT-----GCGGATAACCCTAGTCTGGAG  P864L—GCGGATAACCTTAGTCTGGAG  --------A D N L S L E  -------L R A S T F Y  WT----CTGCGCGCCTCCACTTTCTAC  S943F—CTGCGCGCCTTCACTTTCTAC  -------L R A F T F Y | -------D D F G V P W V D  Wt -- GATGATTTCGGTGTGCCTTGGGTGGAT  F979Z GATGATTT-----------GGGTGGAT  -------D D L-------------G G  -------T V Q A F G E N  WT----ACAGTTCAGGCATTCGGCGAAAAC  AFG---ACAGTTCAGGAATTCTTCGAAAAC  -------T V Q E F F E N | ---------T L A S K K I P  Wt------ACGTTGGCTAGCAAAAAAATACCC  K1009X--ACGTTGGCTAGCTAAAAAATACCC  ---------T L A S stop |
| --- | --- | --- |

**B** 🡨--Acidic domain -🡪 🡨---------------PEHE Domain-------------------------

P864L 🡨----Core --🡪

melanogaster SRFRTATFPYSTRTWEDQEFHCDNEFFLEEADELLADNPS----------LEIPKWRDVPVPPSS----DKIDTELLSDATFERRHQKYVKDEVDRKCRDARYMKEQI

erecta SRFRTATFSYSTRTWEDQEFHCDNEFFLEEADELLADNPS----------LEIPKWRDVPVPPSS----DKTNTEPLSDATFERRHQKYVKDEVDRKCRDARYMKEQI

ananassae SLYRTAAYPYTTRSWEDQEFHCDNEFFLEEADELLADNPS----------LEIPKWRDDPVPPSS----DKKGIEPLSDADFIRRHEPYVKDEIKRKRLDARNLKDML

pseudo SRYRTVLFPYTTRSWEDQEFHRDNEFFDEEADELLSDHPS----------LEIPKWRDTPIPPST----DNKDIESLTDEDFVKRHEKYVKDEIERKRRDARYMREQM

virilis SKFRTSLQPYTTRSWEDQEFHCDNEFFLEEADELLADNPS----------LEIPKWKVVHITRSA----DSKGTEPLSDADFVRRHEKYVRDEIERKKRDARYLREQM

grimshawi -DSTLISEPYTTRSWEDQEFHCDNEFFLEEADELLADNPS----------LEIPKWKMISLRRSS----DDKEIEPMSNDAFEHRHDKYVREEIERKKRDARYNQEQN

aedes CKFMMTSKPYITCNWKDEAITNDLDRLLE--NEAAELE--------------VPCWTVIEDNYDSASSSSELTSENISEEVYLKRHAKFELDERRRKKWDVQRIREQK

culex --FMITKKPYITCNWKDEAITNDIERILE--NEAAELE--------------VPCWTVIDDNNDSASNSSELLTENISDEVYMKRHAKFELDERRRKKWDVQRIREQK

anopheles AAYMVTQKQYVSGSWKDDAVTAEIEKLLS--NEAAELE--------------IPSWTVIEDDGDDPAAS-EPSRENISDEAYAKRHTKLEIDERRRKKWDVQRIREQK

nasonia ----TTEEHYYTAVGEANYALALKDRSSPEANSKS---------------LEVPNWRVKVYTSCY----TMEGTENLDDEIFNKRHLKLENDERRRKRWDVQRIREQR

bee ESFLTTEEHYYTAVGDLNYTLSLKEPNLVESDTS----------------LEVPNWRIKIYTSCY----TMEGTENLDDEIFNKRHLKLENDERRRKRWDVQRIREQR

lice ILKLKTDSTYYTIIGESDSCWSTGEVEN-FKEEEQ---------------VQVPTWREKPVAPCY----VMEGTENLGDDVFSKRHQRLEIDERRRKRWDIQRIREQN

aphid LSLITADDAYYTCLGNYDPVKDEPVSEKTNSN------------------LEIPSWRVKTYSSWY----SMEGTENMSDDIFESRHYRLEQCEQKRKRWDLQRIRELK

tribolium ----TTTDQYITEAGRTNLRLSNVTDMQEIPNNTN---------------LEVPRWRVKVYASCY----AMEGTENLDDEVYNKRHSRLENDERRRKRWDVQRIREQR

ciona QPVLLTKQYYYKRLSTEQSTCTRIVEQEN-FRDDGTVLN-------------IPTWR-VKVIPLE-NTSTESPIEAIDDESYLKRHSKFEIAEKRRKRWDIQRIREYR

trichoplax LPYRHLDHIMNFCAVSNFQNVPPIKVANKDDDVL------------------VPKWRVCDDSNDMLPEPEEEEAETLSDSDYAKRHWKHELDEKRRKRWDLQRIREEQ

lancet GIMRTSSEYYSSLHQAPPSPVLTVKTPQK--VSKDDLH--------------LPPWRVN--NLTTSSLDEKDFVEDITDDAYNRRHQKLELDEKKRKRWDIQRIREKR

tetradon LPFMSTTEMYLCCWNQPPLS--PLRETSPKKEEEVA----------------IPSWRENHIEPLC-EDPSFVPPELLEDGVYLKRHMKLELDEKRRKRWDIQRIREQR

zebrafish LPYMTTTEMYLCCWE-PPDS--SLQEDCPKKEEDVA----------------IPSWRENIMEPLK-EEDAADLPESLDDSVFLKRHAKLELDEKRRKRWDIQRIREQR

salmon LPFMSTTEMYLCRWHQPPPSPQREPSPSPKKEEVVA----------------IPSWKENSMEPLD-EEAASDIPEMLDDSVFLKRHAKLELDEKRRKRWDIQRIREQR

xenopus LPYLTTKDMYLSRWHQPPPSPLREP--SPKKEESVA----------------IPSWREHHIEPLQ-LAEPCDIPENLDDAVFAKRHAKLELDEKRRKRWDIQRIREQR

Chicken LPYLSTTEMYLCRWHQPPPSPLPLREPSPKKEETVA----------------IPSWRDHVVEPLR-DPNPSDILENLDDSVFSKRHAKLELDEKRRKRWDIQRIREQR

opossum LPYLSTTEMYLCRWHQPPPSPLPLREPSPKKEETVARCLVPSSVAGETSVLAVPSWRDHTVEPLR-DPNPSELLENLDDSVFSKRHAKLELDEKRRKRWDIQRIREQR

human LPYLSTTEMYLCRWHQPPPSPLPLRESSPKKEETVARCLMPSSVAGETSVLAVPSWRDHSVEPLR-DPNPSDLLENLDDSVFSKRHAKLELDEKRRKRWDIQRIREQR

mouse LPYLSTTEMYLCRWHQPPPSPLPLRESSPKKEETVARCLMPSSVAGETSVLAVPSWRDHSVEPLR-DPNPSDILENLDDSVFSKRHAKLELDEKRRKRWDIQRIREQR

horse LPYLSTTEMYLCRWHQPPPSPLPLRESSPKKEETVARCLMPSSVAGETSVLAVPSWRDHSVEPLR-DPNPSDLLENLDDSVFSKRHAKLELDEKRRKRWDIQRIREQR

------------------------------------------- PEHE-------------------------------🡪

S943F AFG F979Z

melanogaster RLEQLRMRRN-------------QDEVLVALDPLRASTFYPLPEDIEAIQFVNEVTVQAFGENVVNMEARD---DFGVPWVDAIEAPTSI------------

erecta RLEQLRMRRN-------------QDEVLVALDPLRASTFYPLPEDIEAIQFVNEVTVQAFGENVVNMEARD---DFGVPWVDAVEAPTSI------------

ananassae RSEAMRSRKI-------------QEEVS-----LPTSTFYPLPHDIEAIQFVDEVPVQAFGENMVNMEARD---DFSLPWLDAVHAQTSI------------

pseudo RSEALRQRHN-------------QDEVLVKLDPLPTSTFYPLPEDIEGVQIVTEIPVQAFGENVVNMEARS---DFSLPWLDSVKALTAI------------

virilis RSEALRLRHN-------------QDEVLIPLDPLPTSTFYPLPEDIEGIQFVTEVPVQAFGENMINLKPESE--HFSLPWLDAVHAPTAD------------

grimshawi KIEALRQRHN-------------QDEVLVPLPPLPTSTFYPLPEDIEIIQFVSEVPVQAFGENMMYPRLQRE--HFILPWLS--------------------

aedes TIERLKKRH-------------LKAD--SDSEQKNIGSFFPSVESIKYVQITEDIPVQAFGELIPILPPSS----FSLPWMASHIDTTSINNFTPSELGPGPLFSAATGIP

culex TIERLKKRH-------------LKTD--PDSEQKNIVSFYPLVDSIKYVQVTDEIPVQAFGELIPILPTSS----FSLPWMGASLDAT--DNIPPTEFGPGPLFSAATGVP

anopheles HIERLKKRQ-------------LKEQPVEQEAQKAITTLYPTVDTLKYVLVTDDVPVQAFGELIPLLPTNGG---FSLPWNQPKPGPSFANPQTSCSTTTSSASTASAGMG

nasonia HIEKLKSRQERQSHHATCYNTNHTGPCPSAPEEESVTTMWPEVDQIQSIQVDTQLPISAFGASVPSFSP-----EFSLPWLINSKL

bee HIEKLKQRQERQNHQATCYNTNHTGPCPSTVEEETVSSLWPDIEQIQSLQVDSQLPVTAFGAPIPSFTPS----EFSLPWSNMSRSNS-RRPKRSTGRRKSTRR

lice HVDKLKQKLNKHQN-------------TQSSSEHPLSSFFPSIDDLEEISIEDGLPVLAFGLEIPFFETS----EFSLPWSLHLQ-------KKQKLSSAS

aphid RVEYLKEGRNKHHSQ-----------KSNDNNKEELTTLLPSPSNIKAIEITDKLPVSAFGSNLHIKKKLEIYKFYHSQWYNDGSQAPKECNIGGLRSSGCSQFYDTM

tribolium VIEKLKQRQERMG----------SG-SRTDEQIESVLSLWPTLDNIKYLEVSDQLPVAAFGSPIPKIAPQV--PEFSLPWLANPALITNKRGSTKRTTVRKKNSKR

ciona YNEKLRQKIL--------------KQDLIKDG--SVETFSPSLFEIDALAVDNCLPVNVFGHCLPHLPVQE----FSLSSSR

trichoplax LKENLRRRSEEKSTS----KTLSKSRKRKSLEEQRITSFDPSVDNIKGICVEDTIPVVAFGSTIPIFPSSE----FSLPWFN---ESKVDMDNDERSTKKRRKSRRNN

lancet QYEKLLMRN-----------MMKKCNSDTTWVQPTIGSFYPEPEEVEHVEVGDTVPVVAFGHPLPCITKSE----FSLPWNVG-SYSS-ARGRRSRGGRGRRKFY

tetradon MFQRLQQRMN------------RKK--VITEAERQLSSFYPDTEDVETIVITPFLPVVAFGRPLPKLSQEN----FELPWLDDRSRCRIEVPKKHTPHRTCRK

zebrafish MLQRLQQRME------------KKKPNVVQESEPEVSSFHPDLENVEAIMVTPFLPVVAFGRPLPNLTPQN----FELPWLDEKSKGRVESQKKQTPHRTCRK

salmon MFQRLQQRMN------------KKK--GIQESEPEVSSFYPDTEDVESIIITPFLPVVAFGRPLPKLTQQN----FDLPWQ--RSRCRIEVPKKHTPHRTCRK

xenopus LLQRLQLRMC------------KKK--GNQESEPEVTSFFPDVDDVESVMITPYLPVVVFGRPLPKITPQN----FELPWLDDRSRCRLEMQKKQTPHRTPRK

Chicken ILQRLQLRMY------------KRK--GIQESEPEVTSFFPEPDDVESLLITPYLPVVAFGRPLPKLTPQN----FELPWLDERSRCRLEVQKKQTPHRTCRK

opossum ILQRLQLRMY------------KKK--GIQESEPEVTSFFPEPDDVESLLITPFLPVVAFGRPLPKLTPQN----FELPWLDERSRCRLEIQKKQTPHRTCRK

human ILQRLQLRMY------------KKK--GIQESEPEVTSFFPEPDDVESLMITPFLPVVAFGRPLPKLTPQN----FELPWLDERSRCRLEIQKKQTPHRTCRK

mouse ILQRLQLRMY------------KKK--GIQESEPEVTSFFPEPDDVESLLITPFLPVVAFGRPLPKLAPQN----FELPWLDERSRCRLEIQKKHTPHRTCRK

horse ILQRLQLRMY------------KKK--GIQESEPEVTSFFPEPDDVESLMITPFLPVVAFGRPLPKLTPQN----FELPWLDERSRCRLEIQKKQTPHRTCRK

🡨-------------Cter Domain ------------🡪

K1009X

melanogaster ARSKALAEPVATLASKKIPTTAAEARHQENHSSYVFPKRRKRQKNR 1039

erecta ARSKALAEPVATLASKKLPTTAAEARHQENHSSYVFPKRRKRQKNR

pseudo ARAKAEAVPVATLASKKIPLTAAESRHQEMNSSYVFLKRRKRQRKR

virilis ARAKAQAAPVATLASKKLPTTAAEARHQEMNSSYVFLKRRKRQRRR

ananassae GKAKALAVPVATLASKKLPTNAKEARHQEMNSSYVYLKRRKRPRQR

grimshawi TAATATTATVATLANKRLPTSKKEARHQELNSSYVFLKRRKRQQRR

aedes -----STSTSSVSSSTSHEIKTKFMHRLAPNLIVQKQRFTKRIKKE

culex -----ATATTSAAS---QPVKTKFLHRLAPNLVTQKQRFTKRIKKE

anopheles -----HHGPGPSGLGLLLETKTKFIHRLAPSLQAQKQRFTKRIKKDP

Figure S3. Sequence alignment of MSL1 C-terminus. A. The sequence changes for all mutants in this study are shown. B. A single MSL1-like protein was retrieved from the whole genome sequences of diverse organisms. The Drosophila *NSL1* gene (*CG4699*) was previously reported to contain a PEHE motif (Marin 2003; Mendjan et al. 2006), but we found so little primary sequence similarity to MSL1 that it is not included in this comparison. In a few instances the amino acid sequence predicted in databases differed from the consensus at highly conserved positions, while sequence in another reading frame matched well. In these few instances, the more conserved amino acid prediction is shown. While all twelve available Drosophila sequences were examined, only a diverse subset of species is presented for space considerations. Gray shading indicates strong conservation, and yellow shading indicates Drosophila-specific changes at otherwise highly conserved positions that may provide specialized adaptations for dosage compensation. Purple shading indicates tryptophan residues that serve as highly conserved landmarks. The AFG triplet is shaded turquoise. The five mutants reported in this paper are shaded red.
