# Supplementary material for: A new strategy for isolating genes controlling dosage compensation in Drosophila using a simple epigenetic mosaic eye phenotype
Source: BMC Biol. 2010 Jun 10;8:80. doi: 10.1186/1741-7007-8-80 (PMC2893135; doi:10.1186/1741-7007-8-80)
Supplement: Additional file 3 — Figure S3. roX1 accumulation in msl1 mutants. [file 1741-7007-8-80-S3.DOC]

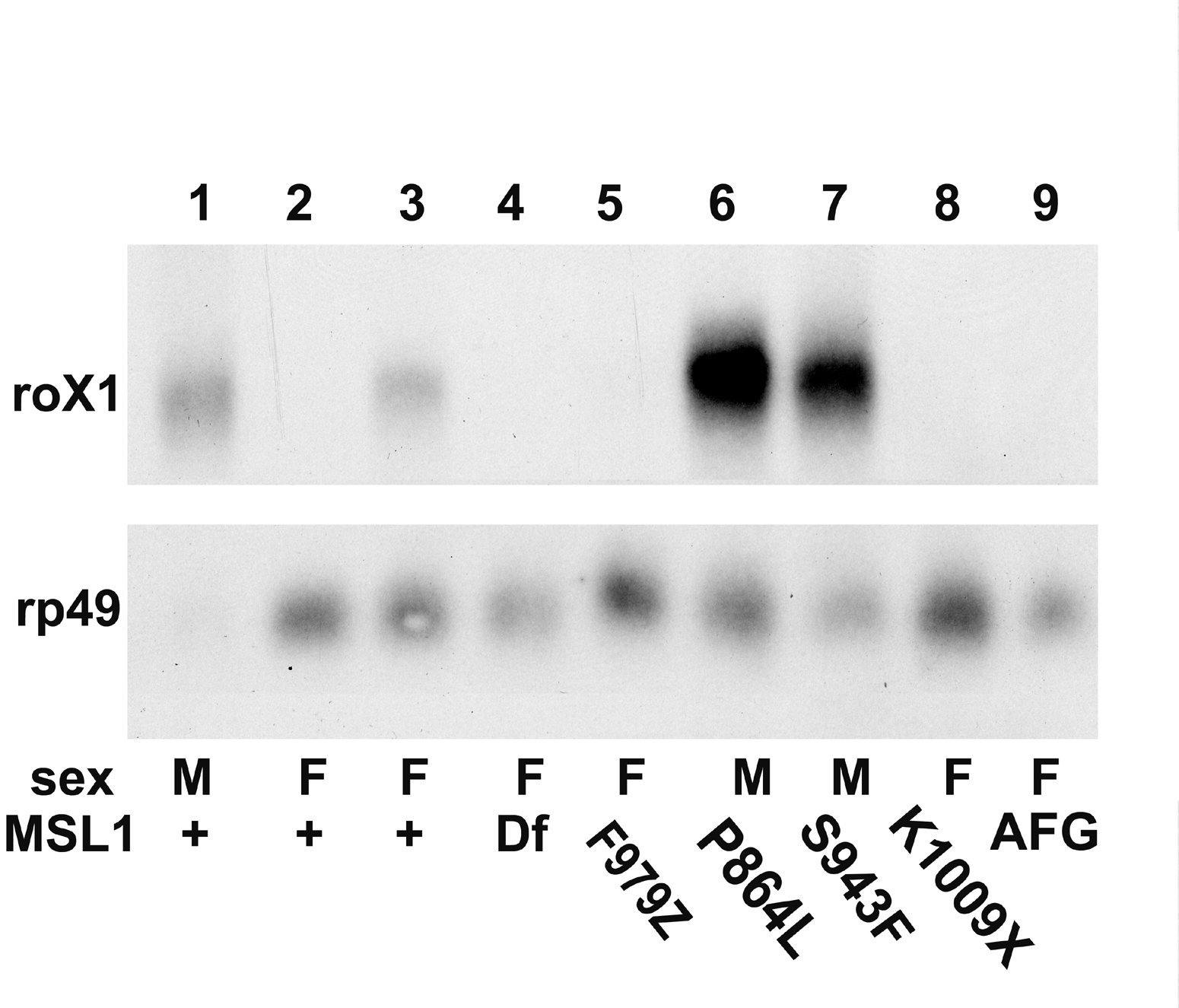


Figure S3. *roX1* accumulation in *msl1* mutants. Total RNA from 1. wild type adult males, 2. wild type adult females, 3. *msl1*^L60^/+; [*w*^+^ *H83M2*] adult females ectopically expressing MSL2, 4. *msl1*^L60^/ *msl1*^L60^; [*w*^+^ *H83M2*] adult females, 5. *msl1*^F979Z^ /*msl1*^F979Z^; [*w*^+^ *H83M2*] adult females, 6. *msl1*^P864L^ /*msl1*^L60^ male larvae, 7. *msl1*^S943F^ /*msl1*^L60^ male larvae, 8. *msl1*^K1009X^ /*msl1*^K1009X^; [*w*^+^ *H83M2*] adult females, 9. *msl1*^AFG^ /*msl1*^AFG^; [*w*^+^ *H83M2*] adult females. The membrane was hybridized with probes for *roX1* and *rp49* as a loading control. The extremely weak *roX1* band in F979Z lane was detectable on a replicate experiment, but no signal was found in K1009X or AFG on prolonged exposures.
